# Supplementary material for: Demonstration of measurement-free universal logical quantum computation
Source: Nat Commun. 2026 Jan 26;17:995. doi: 10.1038/s41467-026-68533-x (PMC12848042; doi:10.1038/s41467-026-68533-x)
Supplement: Supplementary file 1 — Supplementary Information [file 41467_2026_68533_MOESM1_ESM.pdf]

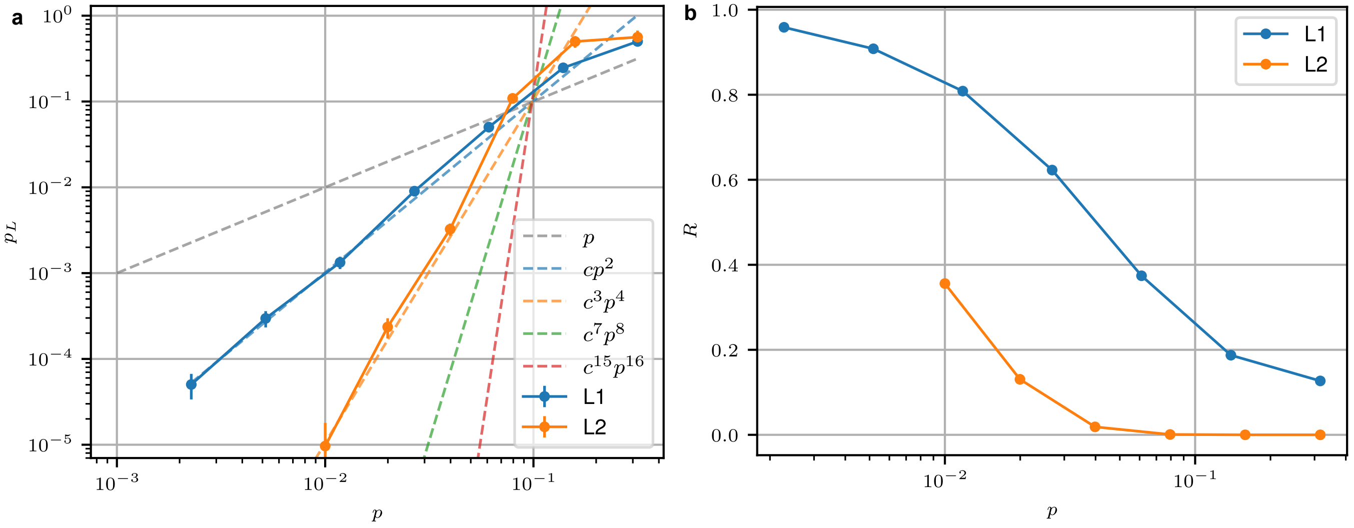

Supplementary Figure 1: **Logical failure rates p_L and acceptance rates R for measurement-free state teleportation with concatenated [[4,1,2]]-codes.** (a) Numerically simulated logical error rates, averaged over logical input states $|0\rangle_L$ and $|+\rangle_L$, for teleportation of a state encoded in a bare [[4,1,2]]-code (L1, solid blue) and the concatenated [[4,1,2]]-code (L2, solid orange). The dashed lines correspond to the estimated error polynomials $p_L^{(n)} = c^{2^n-1} \cdot p^{2^n}$ that approximate the logical failure rate at concatenation level n. The coefficient c was determined by means of fault-path counting. (b) Accepted fraction of runs R determined from numerical simulations for concatenation levels L1 and L2.

Operation	Error rate	Duration
Two-qubit gate	$p_{2q} = 0.025$	350 µs
Single-qubit gate	$p_{1q} = 0.0036$	70 µs
Measurement	$p_{\text{meas}} = 0.003$	-
Preparation	$p_{\text{init}} = 0.003$	-

Supplementary Table 1: **Error rates and duration of operations on a trapped-ion quantum processor.** These values correspond to the trapped-ion setup that was used in the experiments and are used in the numerical simulations. Furthermore, the coherence time is determined to be $T_2 = 50$ ms in our experimental setup.

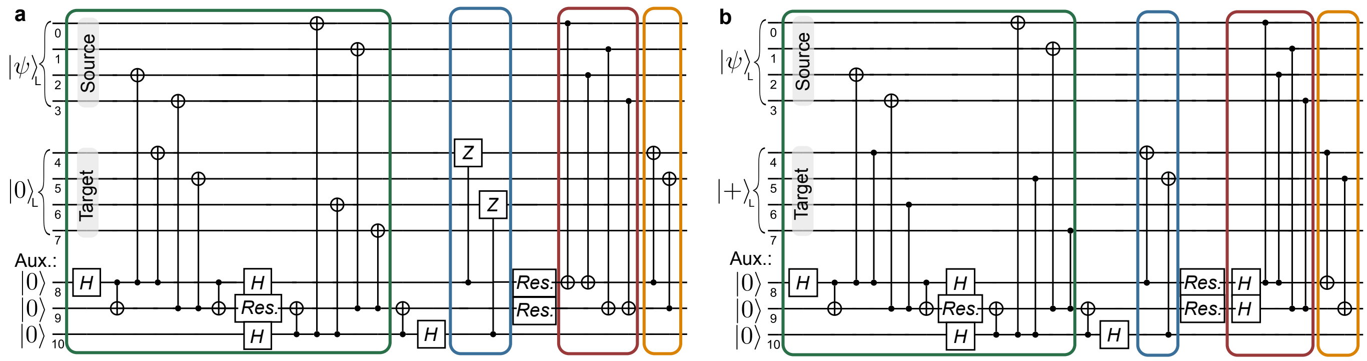

Supplementary Figure 2: **Circuits for modular measurement-free logical state teleportation from a source to the target register and the application of a H_{L}-gate by means of teleportation.** **a,** We first map two stabilizer-equivalent joint logical operators $X_{\mathrm{L}}^{\mathrm{S}}X_{\mathrm{L}}^{\mathrm{T}} = X_2X_3X_4X_5$ and $X_{\mathrm{L}}^{\mathrm{S}}X_{\mathrm{L}}^{\mathrm{T}} = X_0X_1X_6X_7$ that have fully disjoint support onto auxiliary two-qubit GHZ-states (green). Then, two CZ-gates are applied implementing the desired $Z_{\mathrm{L}}^{\mathrm{T}}$, if both values of the joint logical operators are in 1 (blue). Here, *Res.* corresponds to a reset operation as described in the Methods Section, which can either be carried out explicitly by physically resetting the auxiliary qubits to the $|0\rangle$ state and reusing them afterwards, or implemented by replacing them with fresh qubits. In the second step, we map two equivalent operators $Z_{\mathrm{L}}^{\mathrm{S}} = Z_0Z_2$ and $Z_{\mathrm{L}}^{\mathrm{S}} = Z_1Z_3$ onto physical auxiliary qubits (red) and implement two CNOT-gates to apply $X_{\mathrm{L}}^{\mathrm{T}}$, if both auxiliary qubits are in the $|1\rangle$-state (orange). **b,** Analog circuit for applying a H_{L}-gate to the [[4, 1, 2]]-code. We now prepare the target register in $|+\rangle_{\mathrm{L}}$, instead of $|0\rangle_{\mathrm{L}}$. Then we map out two disjoint, but equivalent operators $X_{\mathrm{L}}^{\mathrm{S}}Z_{\mathrm{L}}^{\mathrm{T}}$ (green), implement a coherent quantum feedback with CNOT-gates (blue), map two $Z_{\mathrm{L}}^{\mathrm{S}}$ to physical auxiliary qubits (red) and apply a coherent feedback operation with two CNOT-gates (orange). Note that the last two steps are recompiled into CZ- and CNOT-gates and some H-gates acting on the auxiliary qubits at the end are omitted, as the auxiliary qubits are disentangled from the data-qubit registers and discarded afterwards.

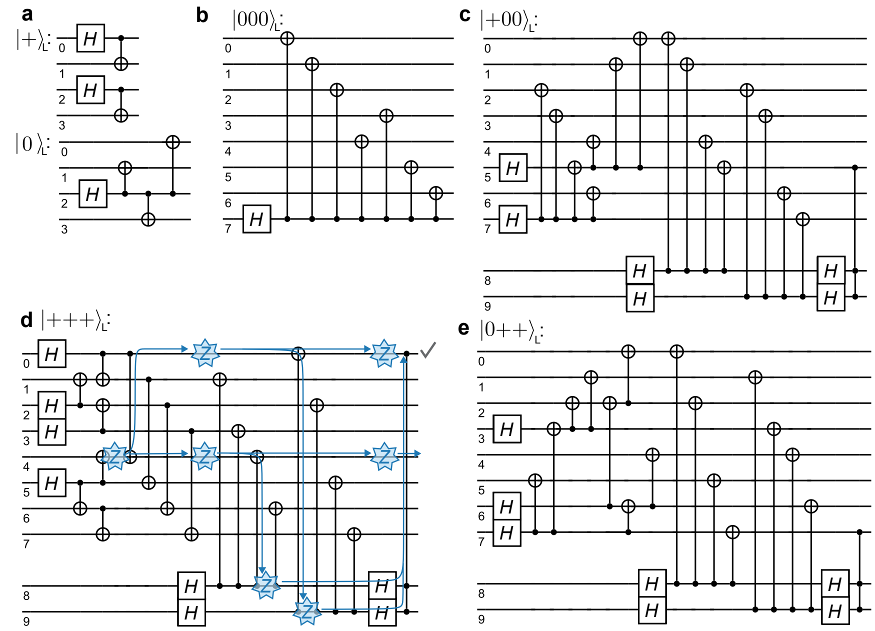

Supplementary Figure 3: **Circuits for fault-tolerant logical state preparation on the** $[[4,1,2]]$ **and** $[[8,3,2]]$ **code. a,** FT initialization circuit for $|+\rangle_\mathrm{L}$ (top) and $|0\rangle_\mathrm{L}$ (bottom) for the $[[4,1,2]]$-code. The first circuit is also used to initialize $|+0\rangle_\mathrm{L}$ on the $[[4,2,2]]$-code. **b-e,** FT initialization circuits for $|000\rangle_\mathrm{L}$, $|+00\rangle_\mathrm{L}$, $|{+}{+}{+}\rangle_\mathrm{L}$ and $|0{+}{+}\rangle_\mathrm{L}$ for the $[[8,3,2]]$-code. For the last three, we add a coherent correction step in the end: two equivalent logical X_L-operators that have fully disjoint support are mapped onto two physical auxiliary qubits, and a CCZ-operation is applied to correct a potentially dangerous weight-2 error resulting from a single fault into a detectable weight-1 error. For example, a single Z-fault in the circuit for the $|{+}{+}{+}\rangle_\mathrm{L}$ initialization (**d**) on qubit 4 (marked in blue) may propagate onto qubits 0 and 4, which directly corresponds to a logical error Z_L^1. In the measurement-free verification step, this error configuration propagates further onto *both* auxiliary qubits. The CCZ-gate in the end applies Z_0 to the data qubits, as both auxiliary qubits have been flipped, effectively removing one of the errors and leaving the detectable error configuration Z_4.

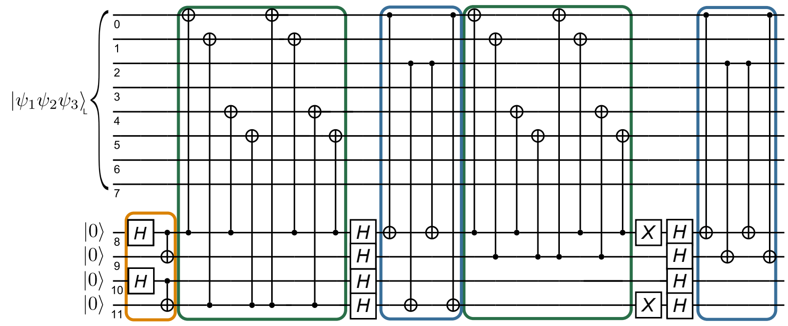

Supplementary Figure 4: **Circuit for a single-logical qubit** H_L**-gate on the** $[[8,3,2]]$ **code, as illustrated in Fig. 2b3.** We first prepare the auxiliary register in the $|+0\rangle_\mathrm{L}$-state of the $[[4,2,2]]$-code (orange). Then, we apply the inter-block CNOT-gate (green) where the control-bit corresponds to the first qubit of the $[[4,2,2]]$-block, and the target qubit to the first qubit of the $[[8,3,2]]$-code. The inter-block CNOT-gate is not transversal but FT in the sense that any single fault may propagate, but is still detectable in the end. In the next step, the $H_\mathrm{L}^{\otimes 2}$-gate is applied to the auxiliary register. This step includes an additional SWAP operation, which is absorbed by the following gates, as the physical qubits are simply relabeled accordingly. We then apply an inverted CNOT-gate (blue), that is controlled by the first logical qubit of the $[[8,3,2]]$ and acts on the first one of the $[[4,2,2]]$-code. The two inter-block logical CNOT-gates are not symmetric, but the implementation depends on the orientation of the gate. After again applying $\mathrm{C_{aux,1}NOT_{\psi 1}}$ (green), and $H_\mathrm{L}^{\otimes 2}X_\mathrm{L}^1$ to the logical auxiliary qubits, we finally apply the last logical gate $\mathrm{C_{\psi 1}NOT_{aux1}}$ (blue).

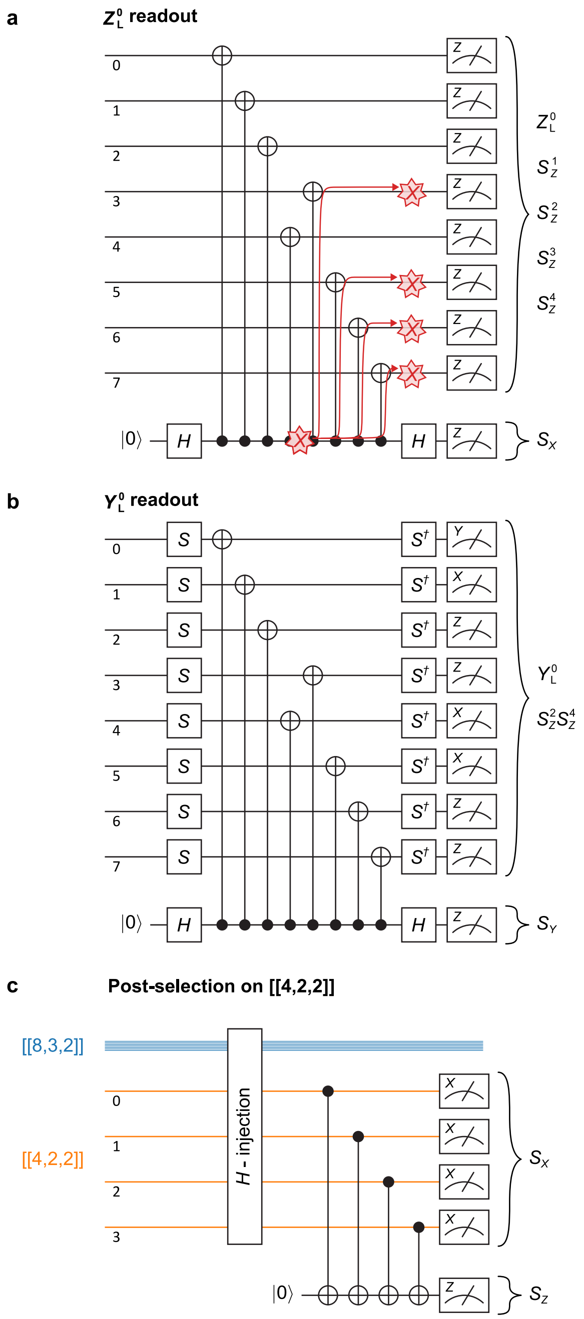

Supplementary Figure 5: **FT measurement instructions for the** $[[8,3,2]]$**-code. a,** Circuit for the FT mapping of the S_X-stabilizer onto a physical auxiliary qubit, required for FT measurements in the Z-basis. A single X-fault on the auxiliary qubit may propagate to a weight-4 error configuration, as for example to $X_3X_5X_6X_7$. This error is detected by e.g. the Z-stabilizer $S_Z^1 = Z_0Z_1Z_2Z_3$. **b,** Exemplary circuit for FT measurement in the Y-basis. We first map the Y-stabilizer S_Y onto a physical auxiliary qubit and subsequently measure the physical data qubits in different bases, as indicated, to infer Y_L^0. **c,** Circuit for stabilizer extraction on the logical auxiliary $[[4,2,2]]$-code. We map the Z-stabilizer onto an auxiliary qubit and then destructively measure the physical qubits in the X-basis.

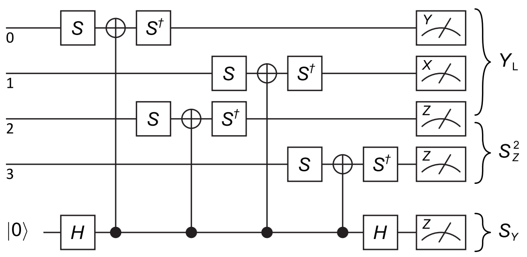

Supplementary Figure 6: **FT measurement of Y_L.** We first map the Y-stabilizer onto an auxiliary qubit and then measure the physical qubits in the Y-, X- and Z-basis to fault-tolerantly measure Y_L.
